# AaTs-1: A Tetrapeptide from *Androctonus australis* Scorpion Venom, Inhibiting U87 Glioblastoma Cells Proliferation by p53 and FPRL-1 Up-Regulations

**DOI:** 10.3390/molecules26247610

**Published:** 2021-12-15

**Authors:** Dorra Aissaoui-Zid, Mohamed-Chiheb Saada, Wassim Moslah, Marie Potier-Cartereau, Aude Lemettre, Houcemeddine Othman, Marc Gaysinski, Zaineb Abdelkafi-Koubaa, Soumaya Souid, Naziha Marrakchi, Christophe Vandier, Khadija Essafi-Benkhadir, Najet Srairi-Abid

**Affiliations:** 1 Laboratory of Biomolecules, Venoms and Theranostic Applications, LR20IPT01, Institut Pasteur of Tunis, University of Tunis El Manar, Tunis 1002, Tunisia; chihebsaada@gmail.com (M.-C.S.); wassim.moslah@gmail.com (W.M.); houcemoo@gmail.com (H.O.); abdelkafi_zaineb@yahoo.fr (Z.A.-K.); marrakchi_naziha@yahoo.fr (N.M.); 2 N2C UMR 1069, INSERM, University of Tours, 37032 Tours, France; marie.potier-cartereau@univ-tours.fr (M.P.-C.); aude.lemettre@univ-tours.fr (A.L.); christophe.vandier@univ-tours.fr (C.V.); 3 Sydney Brenner Institute for Molecular Bioscience, Faculty of Health Sciences, University of the Witwatersrand, Johannesburg 2041, South Africa; 4 Chemistry Technological Platform NMR Department, Faculty of Sciences, University of Nice Sophia Antipolis, Parc Valrose, 06108 Nice, France; marc.gaysinski@unice.fr; 5 Laboratory of Molecular Epidemiology and Experimental Pathology Applied to Infectious Diseases, LR16IPT04, Institut Pasteur of Tunis, University of Tunis El Manar, Tunis 1002, Tunisia; souid@ulm.edu (S.S.); khadija.essafi@pasteur.tn (K.E.-B.); 6 School of Basic Pharmaceutical and Toxicological Sciences, College of Pharmacy, University of Louisiana, Monroe, LA 71201, USA

**Keywords:** glioblastoma, scorpion venom, AaTs-1, antiproliferative effect, FPRL-1 receptor, antagonist

## Abstract

Glioblastoma is an aggressive cancer, against which medical professionals are still quite helpless, due to its resistance to current treatments. Scorpion toxins have been proposed as a promising alternative for the development of effective targeted glioblastoma therapy and diagnostic. However, the exploitation of the long peptides could present disadvantages. In this work, we identified and synthetized AaTs-1, the first tetrapeptide from *Androctonus australis* scorpion venom (Aa), which exhibited an antiproliferative effect specifically against human glioblastoma cells. Both the native and synthetic AaTs-1 were endowed with the same inhibiting effect on the proliferation of U87 cells with an IC_50_ of 0.56 mM. Interestingly, AaTs-1 was about two times more active than the anti-glioblastoma conventional chemotherapeutic drug, temozolomide (TMZ), and enhanced its efficacy on U87 cells. AaTs-1 showed a significant similarity with the synthetic peptide WKYMVm, an agonist of a G-coupled formyl-peptide receptor, FPRL-1, known to be involved in the proliferation of glioma cells. Interestingly, the tetrapeptide triggered the dephosphorylation of ERK, p38, and JNK kinases. It also enhanced the expression of p53 and FPRL-1, likely leading to the inhibition of the store operated calcium entry. Overall, our work uncovered AaTs-1 as a first natural potential FPRL-1 antagonist, which could be proposed as a promising target to develop new generation of innovative molecules used alone or in combination with TMZ to improve glioblastoma treatment response. Its chemical synthesis in non-limiting quantity represents a valuable advantage to design and develop low-cost active analogues to treat glioblastoma cancer.

## 1. Introduction

Glioblastoma is the most aggressive primary brain tumor, reaching 50–60% of gliomas. It is classified as grade IV based on its level of malignancy by the World Health Organization [1]. Despite major advancements in cancer treatment, glioblastoma remains incurable. In fact, after surgical resection, radiotherapy and chemotherapy, the tumor infiltrating glial cells will give rise to tumor recurrence in 94% of cases [2].

Currently, the standard treatment is the protocol “Stupp”, which involves surgery as a first step. However, in some cases, surgical resection causes serious substantial damage and generally fails to remove the entire tumor that infiltrates the normal brain parenchyma. Radiation therapy associated to daily chemotherapy is the second line of standard treatment for patients less than 70 years [3]. The benefit of this combined treatment, in terms of survival, was clearly demonstrated, but still modest and did not exceed 15 months with severe side effects, especially on bone marrow and liver [4,5]. Moreover, the use of conventional chemotherapy, such as temozolomide (TMZ), an oral DNA alkylating agent, is ineffective in 50% of TMZ treated patients due to the intrinsic or acquired drug resistance [6,7]. Over-expression of O^6^-methylguanine methyltransferase (MGMT) and/or lack of a DNA repair pathway in multiple glioblastoma multiforme (GBM) cell lines [8] are common features of refractoriness or acquired resistance of patients to TMZ chemotherapy. Therefore, there is an urgent need to find or produce more effective drugs against glioblastoma [9].

In this context, scientists are currently focused on searching for active biomolecules to develop targeted therapies. Scorpion toxins, such as Chlorotoxin (Cltx), purified from *Leiurus quinquestriatus* scorpion venom [10], or its analogs, have proven particularly useful in pharmacological studies against glioblastoma. Cltx is a short toxin of 40 amino acids [10] that inhibits the migration and invasion of tumor cells in vitro and in vivo [11]. Currently, only TM-601, a synthetic version of chlorotoxin, has reached the phase III clinical trials [12]. It crosses the blood-brain barrier and binds to malignant brain tumor cells, without affecting healthy tissue [12]. The target of Cltx appears to be a protein complex that contains ClC-3 channels and the matrix metalloproteinase 2 (MMP-2). The Cltx binding induces the endocytosis of this complex [13]. Chi Hang Lui et al. have shown that Cltx acted, not only by reducing the expression of MMP-2, but also by inhibiting its enzymatic activity [14]. Previously, our team identified AaCtx, a chlorotoxin-like peptide from *Androctonus australis* scorpion venom (3.6 kDa), which was also effective against glioblastoma cancer cells [15].

However, the synthesis of these toxins has limitations because of their long sequence and the presence of four disulfide bonds, which most often lead to misfolded proteins due to the fast kinetics of folding and the conformational heterogeneity [16]. Besides, the synthetic peptides are often less active than the native ones [17].

Hence, identifying low molecular weight peptides active on glioblastoma cells, would be promising tools to develop efficient anti-glioblastoma molecules. In the present study, we report the identification and characterization of a four amino-acids peptide from *Androctonus australis* scorpion venom, which we named *Androctonus australis* Tetrascorpin-1 (AaTs-1), bearing antiproliferative activity against human U87 glioblastoma cells.

## 2. Results

### 2.1. Purification

**Figure 1:**
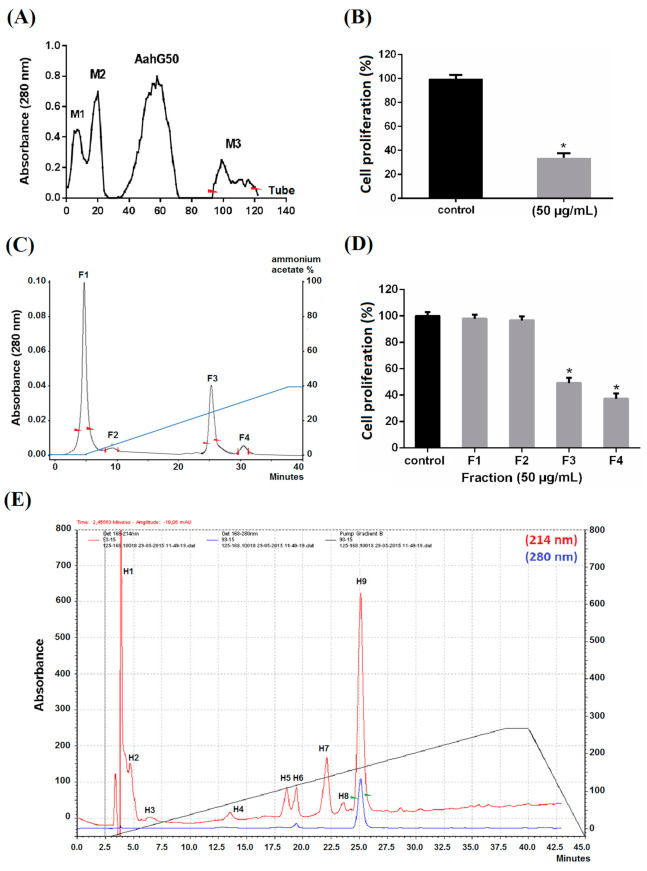
Purification of short bioactive peptides from Aa venom. (**A**) Isolation of the low molecular weight fraction of Aa venom. The crude venom was filtered through Sephadex G-50 gel. Elution was performed with acetic acid (0.1 M) at a flow rate of 0.5 mL/min. The absorbance was measured at 280 nm. Fractions are collected at 2.5 mL/tube. (**B**) Effect of M3 on U87 cell proliferation. The cells were incubated with 50 μg/mL of M3 for 72 h. Mean, SD. * *p* < 0.05, significantly different from control. (**C**) Fast liquid chromatography (FPLC) of M3. The M3 was fractionated by cation exchange chromatography using a Resource-S column. Elution was performed with ammonium acetate (0.5 M) at 0.8 mL/min. The absorbance was measured at 280 nm. (**D**) Effect of FPLC fractions on U87 cell proliferation. The cells were incubated with 50 μg/mL of F1, F2, F3, or F4 for 72 h. Mean, SD. * *p* < 0.05, significantly different from control. (**E**) The fraction F3 produced by FPLC was purified by High Performance Liquid Chromatography (HPLC) on a C18 column. Elution was carried out with a linear gradient (34 min) from 0% to 30% of 0.1% TFA/acetonitrile. The flow rate was 0.8 mL/min. The absorbance was measured at 214 and 280 nm. Peak range collected was indicated by arrows.

In this work, we were interested in small molecules from Aa scorpion venom. Thus, the fraction M3 retained and eluted lastly by the Sephadex G50 chromatography column (Figure 1A) was chosen because it would contain molecules of low molecular masses. We first confirmed that this fraction had an inhibitory effect on the proliferation of human glioblastoma U87 cells. Indeed, the M3 fraction, at 50 μg/mL, showed a 66% inhibition of the proliferation of U87 cells (Figure 1B). The M3 was then fractionated by a Resource S cation exchange column, leading to four fractions (FI to F4) (Figure 1C). When tested at 50 μg/mL, F1 and F2 showed no effect on the proliferation of U87 cells, whereas F3 and F4 fractions exhibited significant inhibitory effects, of 54% and 62%, respectively (Figure 1D). Since F4 represents only 2% of the Aa venom, the major and active F3 fraction, representing 30% of the M3, was fractionated by RP-HPLC. The hydrophobic and major fraction H9 corresponding to peak 9 was eluted at 22 min (Figure 1E). Based on the fact that it represents 72.4% of the F3 fraction and 0.87% of the Aa venom, H9 was chosen to be further studied. 

H9 was first injected intracerebro-ventricularly, as a common route in toxicity assay for scorpion toxins molecules, to Swiss mice (*n* = 6/group). We found that H9 did not induce mice mortality up to 3.5 µg doses (Supplementary Materials Table S1). This amount is 570 times the LD_50_ of AaHII [18], the most toxic peptide of Aa venom, suggesting that H9 is not implicated in the toxicity of Aa venom.

### 2.2. Pharmacological Characterization

**Figure 2:**
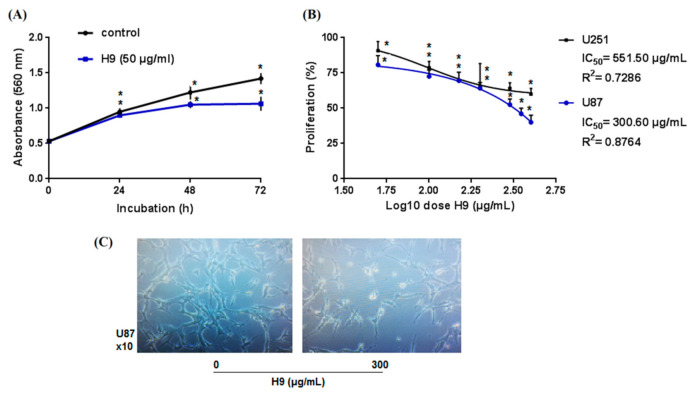
Effect of H9 on glioblastoma cells proliferation. (**A**) To follow the proliferation kinetics, cells are treated with 50 µg/mL of H9 and every 24 h, cells were fixed and stored in PBS to be quantified the last day. (**B**) Dose-response effect of H9 on proliferation of U87 and U251 human glioblastoma cells: cells were treated with different concentration of H9 and incubated for 72 h at 37 °C. The mean, SD. * *p* < 0.05, significantly different from control. (**C**) Microscopic observation of U87 cells after 72 h treatment with 300 μg/mL of H9.

When tested on U87 cells, different concentrations of H9 (50–300 μg/mL) had no effect on the cells’ viability after 24 h. Indeed, cells remained adherent and presented a normal morphology (Supplementary Materials Figure S1). Thereafter, the effect of H9 (50 µg/mL) on the proliferative rate of U87 cells was investigated over three days. Interestingly, the inhibition of cell proliferation was started only after 48 h to reach 25% on the third day (Figure 2A). H9 induced a dose dependent inhibiting effect on the viability of U87 cells after 72 h of treatment with an IC_50_ value of 300 µg/mL (Figure 2B). The IC_50_ value was determined with the GraphPad Prism 6 software by a nonlinear regression analyze using log (concentration) values. Microscopic observations showed that the number of treated cells decreased in comparison to the negative control while maintaining normal adhesion and morphology shape (Figure 2C). 

On the other hand, H9 did not show any inhibitory effect on the adhesion of U87 cells using fibronectin, fibrinogen, collagen I as ECM, or the poly-L-lysine, (Supplementary materials Figure S2A). Additionally, H9 did not exhibit any effect on U87 cell migration or invasion (Supplementary Materials Figure S2B,C) at concentrations up to 300 μg/mL.

H9 was then tested on the proliferation of different cell lines. When tested on U251, another glioblastoma derived cell line, H9 showed less effect than on U87 cells, with only 36% of inhibition at 300 µg/mL (IC_50_ estimated more than 500 µg/mL) (Figure 2B). However, when tested on cell lines derived from other cancer types, such as MDA-MB231 (breast cancer cells), LS174 (colon adenocarcinoma cells), and HepG2 (liver cancer cells), H9 showed no effect (data not shown). 

These results suggest that H9 is selective in inhibiting glioblastoma cell proliferation. 

### 2.3. Structural Characterization and Analysis

#### 2.3.1. Mass Spectrometry Analysis

**Figure 3:**
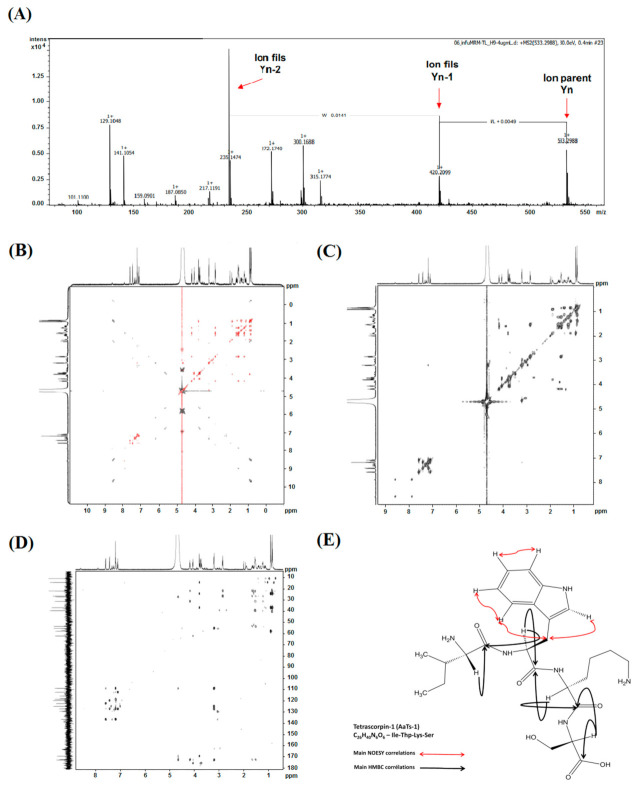
Structural characterization of H9. (**A**) LC-MS/MS spectrum of H9 and amino acid composition. The exact mass of H9 was determined by UHPLC-C18 connected to an ESI-Q-TOF mass spectrometer. 2D NMR spectra: (**B**) ^1^H-^1^H TOCSY, (**C**) ^1^H-^1^H COSY, and (**D**) ^1^H-^13^C HMBC. (**E**) Main NOESY and HMBC correlations for chemical structure of AaTs-1.

The exact mass of H9 was determined by ESI-Q-TOF mass spectrometer. Our results showed that the molecule was pure with an experimental mass of 533.3158 *m*/*z*. Fragmentation, by MS/MS, of mono-charged form (533.29 *m*/*z*) showed that it corresponds to a small peptide of four amino acids (Figure 3A). Manual reconstitution of the amino acids sequence gave two combinations of which the amino acid in N-terminal position is different (I/LWSK). The structural study of H9 was therefore performed by NMR spectroscopy to identify the exact structure.

#### 2.3.2. NMR Analysis

The analysis of NMR data 1D (data not shown) and 2D (^1^H, ^13^C, DEPT135, DEPT90, HSQC, HMBC) identified a structure having 26 carbons (7 Cq sp2 5 CH sp2, 5 CH sp3, 7 CH_2_ sp3, 2 CH_3_ sp3) and 19 protons. TOCSY and COSY experiments allow us to determine the peptidic structure of the compound and to identify the following individual amino acids: serine, tryptophan, isoleucine, and lysine (Figure 3C,D). 

The amino acid isoleucine is characterized by the spin system A_3_MPT(B_3_)X consisting of a δ1-CH_3_ (δH 0.84, δC 10.86), the aliphatic diastereotopic γ1-protonD (γ1Ha: δH 1.13, δC 24.37; γ1Hb: δH 1.39, δC 24.37), the γ2-CH_3_ (δH 0.91, δC14.38), the β-CH (δH 1.89, δC 36.78), and the α-CH (δH 3.8, δC 57.9).

The tryptophan residue is characterized by the indole residue consisting of a η2-CH (δH 7.18, δC 122.38), ζ-CH (ζ3CH: δH 7.09, δC 119.72; ζ2CH: δH 7.42, δC 112.27), the ε3-CH (δH 7.58, δC118.63), the δ1-CH (δH 7.19, δC 124.88), the aliphatic β-CH (δH 3.18, δC 27.35), and the α-CH (δH 4.65, δC 55.25).

The amino acid lysine is characterized by the spin system A_2_(F_2_T_2_)MPX consisting of a ε-CH2 (δH 2.85, δC 39.50), the δ-CH2 (δH 1.54, δC 26.59), the γ-CH2 (δH 1.22, δC 22.06), the aliphatic diastereotopic β-proton (βHa: δH 1.65, δC 31.29; βHb: δH 1.53, δC 31.2), and the α-CH (δH 4.17, δC 53.53).

The amino acid serine is characterized by the spin system AMX consisting of the aliphatic diastereotopic β-proton (βHa: δH 3.77, δC 62.07; βHb: δH 3.71, δC 62.07), and the α-CH (δH 4.05, δC 57.1).

The HMBC experiment allowed us to identify each carbonyl group, i.e., δC=O 175.65 ppm for serine, δC=O 172.43 ppm for lysine, δC=O 172,77 ppm for tryptophan, and δ C=O 169.49 ppm for isoleucine. Connectivity between the different amino acid residues was established through HMBC experiments, i.e., HMBC correlation between α-CH of tryptophan (δH 4.65) α-CH of isoleucine (δH 3.8) and carbonyl moiety of isoleucine amide (δ C=O 169.49 ppm),

HMBC correlation between α-CH of serine (δH 4.05) α-CH of lysine (δH 4.17), and carbonyl moiety of lysine amide (δC=O 172.43 ppm), HMBC correlation between α-CH of lysine (δH 4.17), α-CH of tryptophan (δH 4.65), and carbonyl moiety of tryptophan amide (δC=O 172.77 ppm).

Finally, and despite the use of D_2_O, the TOCSY experiment allowed us to characterize some of the amide and amine proton, i.e., δ N-H proton (δH 10.05 ppm) of indole residue through correlation with the methyne δ1-CH (δH 7.19, δC 124.88), δ N-H amide proton (δH 8.54 ppm) of tryptophan residue through correlation with the α-CH (δH 4.65) the β-proton (δH 3.18), δN-H amide proton (δH 7.92 ppm) of lysine residue through correlation with the α-CH (δH 4.17, δC 53.53) the β-proton (βHa: δH 1.65, βHb: δH 1.53) the γ-CH_2_ (δH 1.22) and the ε-CH_2_ (δH 2.85), δN-H amide proton (δH 7.71 ppm) of sérine residue through correlation with the α-CH (δH 4.05) and the β-proton (βHa: δH 3.77, βHb: δH 3.71) 

Data obtained from the use of the HMBC (Figure 3F) lead to the following concatenation: isoleucine-lysine-tryptophan-serine (IWKS). The chemical structure of H9, which we have named *Androctonus australis* Tetrascorpin-1 (AaTs-1), is presented in Figure 3E. 

The protein sequence data reported in this paper will appear in the UniProt Knowledgebase under the accession number C0HLZ5.

The RMN data has been deposited in BMRbig Biological Magnetic Resonance Data Bank under the title “AaTs-1: a tetrapeptide from Androctonus australis scorpion venom, inhibiting U87 glioblastoma cells proliferation by p53 and FPRL-1 up-regulations -NMR data” and the Entry ID bmrbig30. The main NOESY and HMBC correlations have also been deposited with BMRbig Entry bmrbig31.

#### 2.3.3. Theoretical Analysis of the AaTs-1 Structure 

Sequence analysis of the IWKS showed that AaTs-1 shares 50% identity (and 100% similarity) with Tetrapandin-2 (sequence: LWKT) (Supplementary Materials Figure S3), a tetrapeptide from *Pandinus imperator* scorpion venom. This peptide was reported to be active on a voltage-independent calcium channel, TRPC3, which is a subfamily of the Transient Receptor Potential (TRP) channels [19]. However, the anti-tumor effect of Tetrapandin-2 was never tested on cancer cells. 

On another hand, AaTs-1 had 28% sequence identity with the peptide WKYMVm (Trp-Lys-Tyr-Met-Val-Met-NH2) (Supplementary Materials Figure S3), an agonist for FPRL-1 (human formyl peptide receptor like-1) [20]. 

On the other hand, the Swiss ADME application allowed us to determine the liposolubility of AaTs-1 and to calculate its partition coefficient Kp, which is the ratio of the concentrations of the substance in two immiscible phases (non-polar/aq). In practice, the logarithm of the Log P*o*/*w* partition coefficient (*o*/*w* for octanol/water) is used. AaTs-1 has a log P*o*/*w* value of 2.4, which is close to the optimal value of 2.1 of drugs active on the CNS, and can pass through the blood brain barrier (BBB) [21,22].

### 2.4. AaTs-1 Improves the TMZ Effect on U87 Cells

Based on the fact that a chemotherapeutic drug TMZ, used as a first-line treatment in GBM, exhibited a limited efficacy in some situations, we then asked whether AaTs-1 could enhance its efficacy.

**Figure 4:**
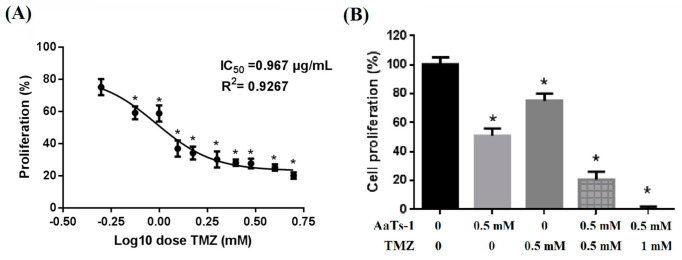
Effect of TMZ on U87 cell proliferation. (**A**) Dose-response effect of TMZ on U87 cell proliferation. Cells were incubated with different concentrations of TMZ (0.5 to 5 mM) for 72 h. (**B**) Co-treatment of U87 cells with TMZ and AaTs-1. U87 cells were incubated with a combination of AaTs-1 (0.5 mM) and TMZ (0.5 mM or 1 mM). Mean, SD. * *p* < 0.05, significantly different from control.

We first evaluate the effect of different concentrations of TMZ (0.5 to 5 mM) on U87 cells proliferation. We found that the effect of TMZ alone on these cells after 72 h, was dose-dependent with an IC_50_ of 1 mM (Figure 4A) determined, with the GraphPad Prisme 6 software, by a nonlinear regression analyze using log (concentration) values. Interestingly, a combination of TMZ (0.5 mM) and AaTs-1 (0.5 mM) induced an increase in the inhibitory effect of cell viability, which exceeded 75% while, used separately, AaTs-1 and TMZ inhibited about 50% and 25% of the cell proliferation, respectively (Figure 4B). When combined at IC_50_ doses (0.5 mM for AaTs-1 and 1 mM for TMZ), the two molecules induced a total abrogation of cell viability (Figure 4B). This result suggests that AaTs-1 sensitizes U87 cells to TMZ.

### 2.5. Analysis of Cellular Responses Involved in the Effect of AaTs-1

**Figure 5:**
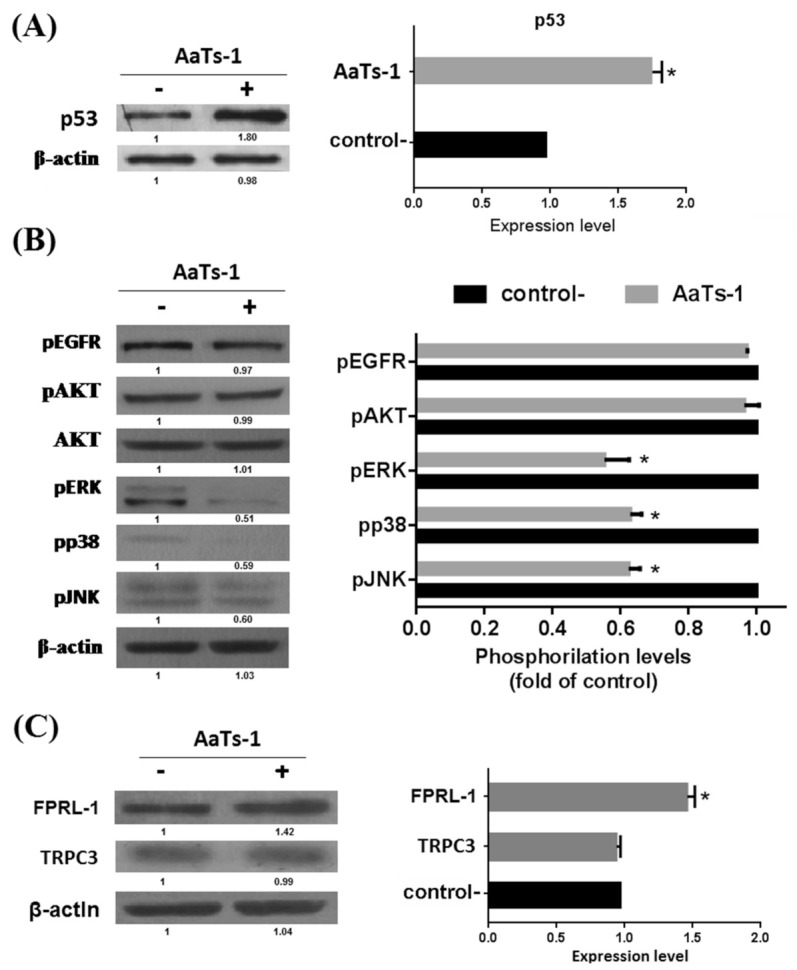
Analysis of cellular effectors targeted by AaTs-1 in U87 cells. Western blot analysis realized with U87 cells lysates treated or not treated (negative control) with AaTs-1 (0.56 mM) using the indicated antibodies (**left**). Tests were done on duplicate minimum. Densitometric representation of the Western blot results (**right**). The graph bars show relative protein expression level normalized to control. Quantitative assay differentiated by Image-J software. Mean, SD. * *p* < 0.05, significantly different from control. (**A**) Analysis of the expression level of p53. (**B**) Analysis of the phosphorylation levels of EGFR; AKT, ERK, p38 and JNK. (**C**) Effect of AaTs-1 on FPRL-1 and TRPC3 protein expression in U87 cells.

To get insight about the mechanism of action of the tetrapeptide, some tests were done.

i. We first tested the effects of AaTs-1 (at the IC_50_ dose = 0.56 mM) on DNA fragmentation. The genomic DNA extracted from mock, SAT (at 1 µM as the positive control), and AaTs-1 treated cells, was visualized on agarose gel electrophoresis. For the positive control, DNA fragmentation was highlighted while a single band appeared in mock- and AaTs-1 treated cells (Supplementary Materials Figure S4A). This indicates that AaTs-1 did not affect the integrity of U87 cell DNA;
ii. Double labelling with Annexin V-FITC/IP of the treated cells was carried out at different time points 24, 48, and 72 h. Flow cytometric analysis (FACS) confirmed that AaTs-1 did not induce apoptosis in U87 cells even after 72 h of treatment (Supplementary Materials Figure S4B);
iii. The cell cycle distribution of the mock-treated cells, stained with propidium iodide (PI) and analyzed by FACS, was also found to be similar to that of AaTs-1 treated cells. The number of cells in the G0/G1, S, and G2/M phases was 7.5%, 64%, and 18.5%, respectively (Supplementary Materials Figure S4C).
  All these results supported that the antiproliferative effect of AaTs-1 on U87 cells is neither due to the induction of apoptosis nor to the cell cycle arrest but rather by another mechanism;
iv. Finally, we investigated the role of some important and studied markers linked to the growth of U87 glioblastoma cells in vitro such as the epidermal growth factor receptor (EGFR) [23], the tumor suppressor protein p53 [24,25], AKT (Protein kinase B); ERK1/2 (Extracellular-signal-Regulated Kinases 1 and 2); p38 (p38 mitogen-activated protein kinase) and JNK (Jun N-terminal Kinases) [26]. Western blot showed that AaTs-1 (0.56 mM) induced a remarkable increase in the expression of p53 protein (Figure 5A) in U87 cells that was 1.8 times higher than that of mock treated cells. Interestingly, the tetrapeptide AaTs-1 decreased ERK, p38 and JNK phosphorylations by 49%, 41%, and 40%, respectively, while no effect was observed on pAKT and pEGFR (Figure 5B).
  Interestingly AsTs-1 was found to induce an increase in the protein level of FPRL-1 by 40% (Figure 5C) and had no effect on TRPC3 protein level (Figure 5C).

### 2.6. Chemical Synthesis of AaTs-1

Given that AaTs-1 represents only 0.87% of the *Androctonus australis* scorpion venom, we chemically synthesized the tetrapeptide on Wang resin by means of Fmoc/But procedure in order to be further studied. 

After preloading the 2-chlorotrityl chloride resin with the first amino acid, stepwise assembly of the synthetic peptides were achieved on Fmoc-Proline-resin by means of optimized Fmoc/t-butyl chemistry [27].

The amount of a target protected peptide linked to the resin was 0.6 mmol, which represents a 75% of yield assembly. 

**Figure 6:**
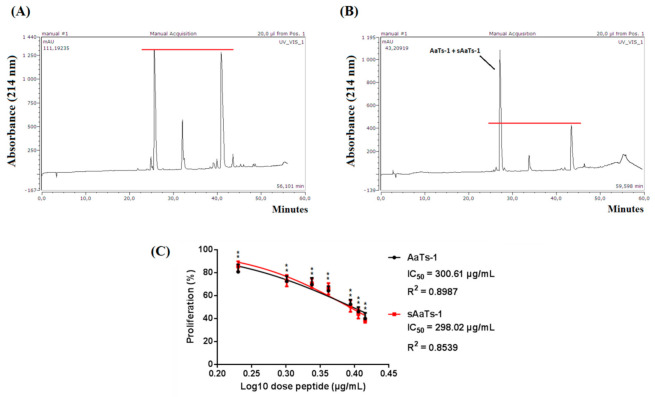
Purification of the synthetic AaTs-1. (**A**) A quantity of 15 μg of the synthesis product was injected into a C18-HPLC column. Elution was carried out with a linear gradient (34 min) from 15% to 30% of 0.1% TFA/acetonitrile. The flow rate was 0.8 mL/min. The absorbance was measured at 214 nm. (**B**) A quantity of 5 μg of the synthesis product was co-injected with an equal amount of the native peptide under the same conditions. (**C**) Dose-response effect of AaTs-1 and sAaTs-1 (50–400 µg/mL) on U87 cell proliferation: The cells were treated with a different concentration of native or synthetic peptide and incubated for 72 h at 37 °C. Mean, SD. * *p* < 0.05, significantly different from control.

The cleaved peptide was precipitated with diethyl ether and then purified by HPLC. The analytical HPLC profile of the synthetic product showed three major peaks representing different forms of synthetic peptides, most likely due to lags in the synthesis (Figure 6A).

In order to identify the peptide with the exact sequence of AaTs-1, the native peptide was co-injected with the construct on an analytical C18 column. Co-injection generated amplification of the first peak at a retention time of 27 min, while the amplitude of the two other peaks did not change (Figure 6B). Thus, the first peptide eluted from the column corresponds to the synthetic form of AaTs-1, which we designated as sAaTs-1. The purification process leads to the recovery of a single peak, indicating that the retention time was identical to that of native AaTs-1 (data not shown).

To confirm the functionality of sAaTs-1, the dose-response proliferation curves were performed in the same conditions to compare the effects of the native and the synthetic peptides on the proliferative of U87 cells. Similar effects were observed, and the results showed that sAaTs-1 has the same dose effect as that of AaTs-1 with the same IC_50_ (0.56 mM) (Figure 6C). Thus, sAaTs-1 was used to study its effect on intracellular calcium release.

### 2.7. Effect of AaTs-1 in Calcium Influx

**Figure 7:**
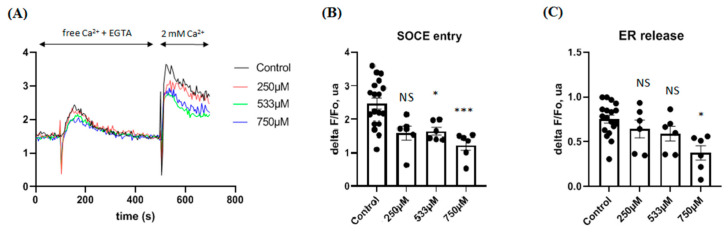
Effects of acute application of sAaTs-1 on the release of Ca^2+^ stores and amplitude of SOCE in U87 cells. (**A**) Cells were stimulated with different doses of sAaTs-1 and have been loaded with fura-2 measuring amplitude of fluorescence ratio variation in comparison to the control condition. (**B**) SOCE entry. (**C**) ER release. Individual values and the mean value +/− SEM of *n* observations (*n* = 3) are presented in the different histograms. Statistical analysis and histograms were created with Prism 8 software. Kruskal-Wallis test and Dunn’s post-test were used with a *p*-value < 0.05 to be considered significantly different to control condition (* *p* < 0.05; *** *p* < 0.001; NS not significant).

Once the potential receptor was identified, the effect on calcium release, which necessitates a huge quantity of the molecule, was verified using the sAaTs-1. We found that the peptide decreased cytosolic calcium concentration in U87 cells. Figure 7 shows that the peptide significantly decreases store-operated Ca^2+^ entry (SOCE) in a dose-dependent manner from 533 µM dose. The effect on the emptying of the endoplasmic reticulum becomes significant for a higher dose (750 µM).

### 2.8. In Silico Study of AaTs-1/FPRL-1 Interaction

We designed a computational analysis to study the putative interaction of AaTs-1 with FPRL-1. The search for a template identified the structure of the anaphylatoxin C5 a receptor (C5AR) (PDB code: 6C1Q) [28] as homologous protein to FPRL-1. While the percentage of identity is only of 35%, the e-value of 10^−51^ and sequence coverage of 92% suggest a strong homology between the FPRL-1 and the template. The structure of the used template is a co-crystal of C5AR with its antagonist anaphylatoxin, PMX53 [29]. Interestingly, this peptide contains a sequence WR, predicting the same structural characteristics of the W_2_K_3_ sequence in the AaTs-1 peptide. Therefore, these two dipeptides were aligned together in the guiding local alignment. The conformations of I_1_ and S_4_ of AaTs-1 were only modeled using the molecular mechanics refinement method of MODELLER. 

**Figure 8:**
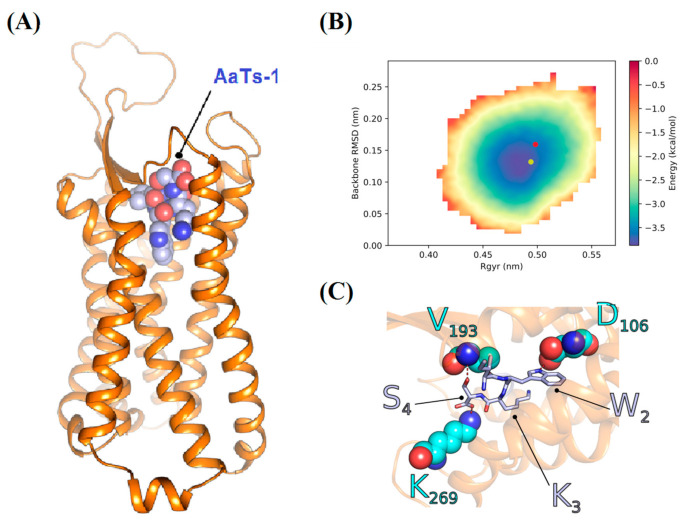
Computational analysis of the interaction of AaTs-1 peptide with FPRL-1. (**A**) Best complex according to the Rosetta score energy describing the interaction of AaTs-1 with FPRL-1. (**B**) Free energy landscape of AaTs-1 calculated from an ensemble of conformations generated with Monte Carlo simulation. The position of the docking conformation and the local minima are marked in red and yellow dots, respectively. (**C**) Polar interactions established between the residues of AaTs-1 and the residues of FPRL-1.

The stereochemical quality of the selected complex of AaTs-1-FPRL-1 was verified by the Ramachandran plot, which indicated only 0.3% of residues in an outline region. The predicted complex was then applied as input structure for simulation. We generated 10 conformations of the complex, which were ranked according to docking score function. We noticed that the set of poses were relatively homogeneous. Indeed, the RMSD (Root Mean Square deviation) calculated on the Cα atoms of AaTs-1 showed that most of the pair wise values were below 2 Å except for two solutions (the 5th and the 10th ranked) which were outliers (Data not shown). This demonstrates that a large fraction of the docking poses could describe the same interaction mode (pose 1–4 and 6–9). We therefore continued our analysis based on the 1^st^ ranked structure (Figure 8A). 

We proceeded with a Monte Carlo peptide folding simulation in order to check if the conformation of the peptide described by the docking solution belongs to a local minimum on the energy landscape. The simulation sampled 10^8^ different conformations. We calculated the RMSD value relative to the initial structure and the gyration radius over all the sampled snapshots and we used them as reaction coordinates to overview the free energy landscape of AaTs-1 (Figure 8B). We noticed the presence of one local minima corresponding to the most stable conformation of AaTs-1 to which the bound conformation with FPRL-1 was very close. The difference of energy levels between the local minima and the bound conformation are less than 1 kcal/mol. We therefore conclude that the bound structure described in the docking corresponds to a stable conformation of the AaTs-1 peptide, which may facilitate the transition of the peptide from its native conformation to the bound conformation, with only minor uphill movements on the free energy landscape. Our predicted complex also highlights the importance of residues W_2_, K_3,_ and S_4_ of AaTs-1, which favorably stabilize the complex by establishing steric contacts and hydrogen bonds with, respectively, D_106_, V_193,_ and K_269_ of FPRL-1 cavity (Figure 8C). Residues W_2_ and K_3_ of AaTs-1 are interacting with the bulky cavity of FPRL-1 by establishing different steric contacts. However, it might also be possible that the charged group of K_3_ is stabilized by hydrogen bonding with water molecules inside the interaction cavity, as there are still some unsatisfied steric contacts. This would also help to neutralize the positive charge of K_3_ and further stabilize the residue. A hydrogen bond is formed between W_3_ of AaTs-1 side chain and D_106_ of FPRL-1 (Figure 8C). Additionally, S_4_ of AaTs-1 is capable to form a hydrogen bond via its side chain hydroxyl group with V_193_ and a salt bridge via the carboxylic C-terminal group with K_269_ (Figure 8C).

## 3. Discussion

In addition to their recurring and highly invasive character, glioblastoma is particularly resistant to actual therapies. To enhance the effectiveness of palliative chemotherapy, clinicians used adjuvant chemotherapy by combining conventional treatment with other drugs [30]. The chemotherapeutic drug temolozolomide (TMZ) is considered as a cornerstone of GBM treatment; however, it is unfortunately also a key factor in tumor resistance and recurrence [31]. Interestingly, the use of TMZ combined to RGD (Arg-Gly-Asp) peptide successfully inhibited the growth of U87 cells in vitro and in vivo, and the cotreatment was effective in the selective delivery of TMZ in glioblastoma cells [32]. Thus, the development of combined treatments constitutes an effective strategy to sensitize refractory cells and to overcome drug resistance [7]. However, the antiproliferative effect of the majority of natural or chemical molecules is generally associated with a cytotoxic effect [33], which is nonspecific and can affect healthy cells. This has prompted researchers to focus specifically on non-cytotoxic molecules acting via non-apoptotic pathways [34], especially as glioblastoma cells are characterized by their resistance to apoptosis [6,7]. Nevertheless, until now, only few molecules have been discovered [34].

In the present work, we have purified and characterized AaTs-1, from *Androctonus australis* (Aa) scorpion venom, able to specifically inhibit the proliferation of human glioblastoma cells via a non-apoptotic mechanism. 

The IC_50_ value of AaTs-1 (0.56 mM) was found to be comparable to that of the RGD peptide [35]. Interestingly, compared to TMZ (IC50 of 1 mM) (Figure 4A), AaTs-1 was nearly twice more active. Furthermore, our results showed that the sensitivity of the U87 cells to TMZ was improved by AaTs-1 and that the combination (Figure 4B) could elicit the advantage to reduce the glioblastoma drug resistance. This effect is most likely due to the original mechanism by which AaTs-1 inhibits U87 cells proliferation. 

Indeed, we showed that the tetrapeptide reduced the proliferative rate of U87 cells without altering their genomic DNA neither by inducing apoptosis nor cell cycle arrest, unlike the most identified antitumor molecules. 

Besides, AaTs-1 enhanced the expression of the p53 and inhibited ERK, p38, and JNK kinases phosphorylation. This may be behind the improvement of U87 cells chemosensitivity. The importance of the tumor suppressor protein p53 in glioblastoma pathogenesis and the role for the MAPK family such as p38, ERK, and JNK kinases in the regulation of a range of cellular functions, including cell proliferation, were well documented [36]. In fact, SGT-53, a nanocomplex that delivers wild-type p53 to tumor cells, improved, in vitro and in vivo, the responsiveness to TMZ treatment in resistant glioblastoma cells [37]. Furthermore, U87 cells that express wild-type TP53 protein [38] are more resistant to TMZ treatment than U251 that express mutant p53 protein [39]. Interestingly, AaTs-1 is more active on U87 cells than U251 cells, suggesting that this molecule is more specific for wild-type p53. The significant increase in the expression of p53 could be explained by the existence of a direct link between this protein and JNK, which is involved in the stability and maintenance of the constitutive level of p53. Indeed, in vitro and in vivo studies have shown that JNK, like MDM2 oncoprotein [40], is a negative regulator of p53 [41,42,43] leading to its ubiquitination and degradation. Therefore, the existence of a cross talk between JNK and p53 can be suggested. Thus, in AaTs-1-U87-treated cells, the decrease in the level of JNK phosphorylation could be linked to the negative regulation of p53 (Figure 5A). 

To get more insights into the key molecular players, we then investigated whether AaTs-1 triggers this downstream cellular response and what could be its first effector/receptor.

In order to identify the target of AaTs-1, we first explored its structure-function relationship. The pharmacokinetic parameters of AaTs-1 showed that its partition coefficient (Log P*o*/*w* = 2.4) is comparable to those of the majority of the central nervous system drugs (1.5 to 2.718) [22]. Thus, the small size of AaTs-1 as well as its theoretical log P*o*/*w* value, close to the optimal value, suggests that the tetrapeptide can easily cross the BBB and reaches the tumor cells by simple diffusion [44].

On another hand, AaTs-1 (IWKS) presented high sequence similarity with tetrapandin-2 (LWKT) isolated from the African *Pandinus imperator* scorpion venom and wasshown to be active on TRPC3 (Transient Receptor Potential 3), a non-voltage dependent calcium channel [45]. However, AaTs-1 did not modify the expression level of TRPC3 in U87 cells (Figure 5C). This result was quietly expected since AaTs-1 was not active on MDA-M231 cells (data not shown), in which this receptor is overexpressed. Furthermore, TRPC3 expression in human malignant gliomas was reported to be in the same range with that of normal brain tissues [46]. 

Besides, AaTs-1 showed 28% sequence identity with the peptide WKYMVm, a specific agonist of the formyl peptide receptor-like1 (FPRL-1), reported to be expressed in U87 cells and promoting their proliferation [47,48]. FPRL-1 is a G protein-coupled receptor (GPCR) reported to also be expressed in U251 glioma cells [28], on which AaTs-1 has also shown an inhibiting effect. 

In order to study the possible interaction between AaTs-1 and the FPRL-1, a model of the AaTs-1-FPRL-1 complex was constructed using the co-crystal of CsAR with its antagonist PMX53 [49], as template. The computational study predicted a stable bound conformation of AaTs-1/FPRL-1 complex (Figure 8), supporting the potential AaTs-1-FPRL-1 interaction.

The comparison of the arrangement of AaTs-1 structure in the complex with that of the antagonist PMX53 co-crystallized with its receptor C5AR, showed that the two ligands have the same disposition on their respective receptors. Indeed, the complex structure showed that the binding of PMX53 to C5AR may restrain the side chain movement of I_116_ and V_286,_ and thereby block the activation switch [49]. Similarly, the model showed that AaTs-1 bind with D_106_, V_193_ and K_269_ of FPRL-1 cavity and could block the receptor. These data suggest that AaTs-1 acts as an antagonist of FPRL-1.

In addition, compared to WKYMVm, an agonist of FPRL-1 [50], AaTs-1 acts differently on the MAPK activation. Indeed, our results showed that AaTs-1 modulated ERK, P38, and JNK dephosphorylation leading to the inhibition of the MAPK pathway (Figure 5B). However, WKYMVm, as well as its analogs have been reported to induce a transient activation of ERK in RBL-2H3 leukemia cells [50]. 

These ascertainments support the hypothesis that AaTs-1 would act as an antagonist, unlike the WKYMVm peptide.

On the other hand, our results showed that AaTs-1 enhanced the expression of FPRL-1 receptor (Figure 5C), while it inhibits U87 cell proliferation. The increase of FPRL-1 expression is most likely due to regulatory mechanisms in the cells following the application of the peptide and that tend to reduce the disruptive effect of the latter over time. In fact, it has been reported that a chronic treatment with an antagonist induces a phenomenon of hypersensitization “up regulation”, which results in an activation of the expression of the receptor [51].

The inhibition of FPRL-1 activity is confirmed, using the synthetic that is also active on U87 cells (Figure 6C), by the decrease of the cytosolic calcium concentration affecting the emptying of the ER on FPR-expressing U87 cells (Figure 7), contrary to WKYMVM, a l-conformer of WKYMVm specific agonist for FPRL-1 [52], that activates the calcium mobilization both in CHO/FPRL-1 and U87 cells via the store-operated channels (SOCs) [20].

In fact, FPRL-1 is a receptor coupled to phospholipase C (PLC), able to mobilize intracellular calcium in U87 astrocytoma cells [20], by the activation of PLC/IP3 pathway [53] and the process of SOCE, which is implicated in the proliferation and tumor progression [54]. The SOCE process, whereby calcium influx across the plasma membrane, is related to the IP3 receptor (IP3R) leading to its activation in response to depletion of intracellular calcium stores of the endoplasmic reticulum (ER) [55]. Our results showed that the blocking of FPRL by AaTs-1 reduced SOCE by acting both on SOC (store operated channels), an effect observed near IC_50_ for AaTs-1, and for higher AaTs-1 concentrations on ER release and most likely on the stromal interaction molecules STIMs (the reticular protein partners connected to SOC) [56]. Both concentrations SOCE were reduced, which could explain the AaTs-1 effect on cell proliferation.

In addition, several studies have reported the direct calcium-dependent activation of many signaling pathways including the ERK pathway [57]. Hence, the activation of PLC is one of the earliest events following binding of various ligands to cell-surface receptors [58] and the metabolites generated control intracellular concentrations of free calcium and protein kinase activity [59].

All these results support the in vitro and in silico AaTs-1 effects, allowing to elucidate the mechanism by which AaTs-1 inhibits the glioblastoma cell proliferation. Therefore, we suggest that AaTs-1 could target FPRL-1 as an antagonist, and thereby induce a decrease in intra-cellular calcium concentration leading to MAPK proteins (ERK/p38/JNK) dephosphorylations associated to increased p53 expression.

The discovery of AaTs-1 may improve the therapy of glioblastoma and pave the way for the design of analogs with better activity and affinity [60,61].

Chemical synthesis of AaTs-1 in non-limiting quantity will represent a valuable advantage to design and develop low-cost active molecules to treat glioblastoma cancer.

## 4. Experimental

### 4.1. Materials

Venoms of *Androctonus australis* scorpion (Aa) from Beni Khedach (Tunisia) were collected by the veterinarian service of the Pasteur Institute’s serpentarium (Tunis, Tunisia) and stored at −20 °C in its crude form until use. 

Swiss mice (20 ± 2 g) were provided by the veterinary service of Pasteur Institute of Tunis.

The U87 glioblastoma cancer cells were generously provided by Dr. José Louis from CNRS UMR 7051 Institut de neuro-physiopathologie, Faculté de Pharmacie (La Timone, Marseille, France).

Cell culture supplements and reagents were purchased from GIBCO (Cergy-Pontoise, France). The extracellular matrix and reagents used for chemical synthesis were obtained from Sigma (St. Louis, MO, USA). Matrigel™ was from BD Biosciences, Pont de Claix, France. Primary and secondary blotting antibodies were from Cell Signaling Technology. Spectra^TM^ Multicoclor Broad Range Protein Ladder was from ThermoScientific. The p53 and TRPC3 antibodies were purchased from R & D Systems at Bio-Techne brand (Minneapolis, MN, USA) and FPRL-1 was from Sigma (St. Louis, MO, USA). Chemicals (reagent grade) were from commercial sources Sigma (St. Louis, MO, USA).

### 4.2. Purification 

Crude venom from Aa scorpion (250 mg) was dissolved in cold water (1:4 *v/v*) and loaded on Sephadex G-50 gel filtration chromatography column (2 × K26/100) (Pharmacia, Uppsala, Sweden) equilibrated and eluted with 0.1 M acetic acid as described by Miranda et al., 1970 [62]. The last eluted fraction, containing small molecules, was lyophilizated and later fractionated by FPLC on cation exchange column (RESOURCE™ S HR 5/5.6 mL, 16 × 30 mm, GE Healthcare) pre-equilibrated with water and operated by Chromeleon software on FPLC (DIONEX Ultimate 3000 FPLC™). Proteins were eluted with a 40 min linear gradient from 0 to 40% of 0.5 M ammonium acetate (pH 8.5) at a flow rate of 0.8 mL/min. Absorbance was monitored at 280 nm.

HPLC purification was performed using a C18 reversed-phase HPLC column (5 µm, 4.6 × 250 mm; Beckman, Fullerton, CA, USA) equipped with a Beckman Series 125 pump and a Beckman diode array 168 detector set. Proteins were eluted from the column at a 0.8 mL/min rate using a linear gradient (30 min) from 0 to 30% of buffer B (0.1% TFA in acetonitrile) in buffer A (0.1% TFA in water) monitored by Karat32 software. Absorbance was monitored at 214 and 280 nm.

### 4.3. Pharmacological Characterization

#### 4.3.1. Intracerebro-Ventricular Toxicity Test on Mice

In vivo toxicity was tested on 20 ± 2 g male Swiss mice by intracerebro-ventricular (ICV) injection of 5 µL of 0.1% (*w/v*) BSA solution containing increasing amounts of the purified peptide. Six mice were used for each dose; and three control mice were injected with only 0.1% BSA in water. ICV administration was performed under ether anesthesia [63]. Survivals were recorded 24 h later. Experiments on mice were carried out in accordance with the European Community Council Directive (2010/63/*EU*) for experimental animal care and all procedures met with the approval of the Institutional Research Board of the Pasteur Institute of Tunis.

#### 4.3.2. Cell Culture

U87 cells were routinely cultured in MEM (Minimum Essential *Medium*) or DMEM (Dulbecco’s Modified Eagle Medium) (Gibco™, Sigma, St. Louis, MO, USA) supplemented with 10% fetal bovine serum (FBS), 1% l-glutamine, and 100 IU/mL penicillin/streptomycin. Cells were maintained at 37 °C in a humid atmosphere of 5% CO_2_.

#### 4.3.3. Cell Viability Test

Cells were incubated with different concentrations of the tested molecule. After 24 h, cells were treated with MTT (0.5 mg/mL). The crystals formed after the reduction of MTT by mitochondrial dehydrogenases were dissolved with DMSO. The quantification of live cells was achieved by measuring absorbance at 560 nm. A negative control was used in the same condition with mock-treated cells. Cells treated with 0.1% Triton X-100 were used as positive control.

#### 4.3.4. Cell Proliferation Assay

Cells were seeded on a microtiter plate (5000 cells/well) and incubated overnight at 37 °C in a humid atmosphere of 5% CO_2_. The medium was renewed in the presence of the purified molecule to be tested. After 72 h of incubation, the wells were washed with PBS before treatment with 1% glutaraldehyde and staining with 0.1% crystal violet. Cells were quantified by reading absorbance at 560 nm.

To track the proliferation kinetics, every 24 h the wells of the day were washed with PBS and the cells were fixed with 1% glutaraldehyde and stored in PBS. The third day the cells were stained with crystal violet 0.1% and quantified by measuring the absorbance at 560 nm. For the determination of IC_50_, the test was done with different concentrations of the active molecule and the results were revealed after 72 h of incubation. 

#### 4.3.5. Cell Adhesion Assay

Adhesion assays were performed as previously described [64]. U87 cells in single cell suspension (10^6^ cells/mL) were treated with 50 µg/mL of the purified peptide, then deposited on wells coated with fibronectin (Fn) at 10 µg/mL, fibrinogen (Fg) at 5 µg/mL, collagen-I (Coll-I) at 50 µg/mL, as extracellular matrix (ECM), or poly-L-lysine (Pl) at 20 µg/mL. Cells were allowed to adhere to the substrata for 2 h at 37 °C. After washing, attached cells were fixed, stained by 0.1% crystal violet, lysed with 1% SDS and quantified by measuring absorbance at 560 nm. 

#### 4.3.6. Cell Migration and Invasion Assays

Cell migration assays were performed using modified Boyden chambers (NeuroProbe Inc., Bethesda, MD, USA) as previously describe [64]. Membranes were coated with Fn (5 µg/mL), for 2 h at 37 °C. Cells were harvested as a single cell suspension (10^6^ cells/mL) and pretreated with 50–300 µg/mL of the purified peptide, then added to pre-coated membranes and allowed to migrate for 5 h at 37 °C in Boyden chambers. Cells were fixed on the underside of the membrane, stained by 0.1% crystal violet and migration was quantified by measuring the absorbance at 560 nm. Cell invasion was also evaluated in Boyden chambers. Matrigel™ was added on the membrane and allowed to solidify 3 h at 37 °C. U87 cells pretreated with the venom peptide were then added and incubated for 22 h at 37 °C. Cell invasion was performed and quantified as above.

### 4.4. Biochemical and Structural Characterization

#### 4.4.1. Mass Spectrometry Analysis

The sample was injected and separated on a thermostatically controlled (35 °C) C18 column (2.1 × 100 mm, 120 Å, Acclaim RSLC, PA2, Thermo Scientific, Waltham, MA, USA), with two successive linear gradients from 2% to 50% (38 min) and from 50% to 70% (10 min) CH 3 CN/0.1% AF, in a U3000 RSLC system (Thermo Scientific, Waltham, MA, USA), at a flow rate of 0.4 mL/min. The UHPLC system was connected to an ESI-Q-TOF mass spectrometer (micro TOFQII, Bruker Daltonics, Billerica, MA, USA). The mass spectrometer was calibrated with a mixture of known masses (ESI-L Low concentration Tuning Mix, Agilent Technologies, Santa Clara, CA, USA). Data were acquired in positive ionization and in MS and MS/MS scan modes. The source temperature was 195 °C, the spray voltage was 5 kV, the nebulizer gas (nitrogen) pressure was set at 2.9 bars and the dry gas (nitrogen) flow rate was 10 L/min. The acquisition of a mass spectrum corresponded to the summing of 4000 scans/s for each spectrum, over a mass range of 50–3000 *m/z*. The LC-MS/MS raw data were processed with Data Analysis (ESI Compass 1.3, Bruker Daltonics, Billerica, MA, USA) software. 

#### 4.4.2. NMR Analysis

High-resolution NMR spectra were recorded in D_2_O at 298 ± 0.1°K (temperature control BCU 05, BVT 3000), on a Avance DRX 500 spectrometer (Bruker, Billerica, MA, USA) operating at 500.03 MHz for ^1^H and 125.76 MHz for ^13^C. One-dimensional NMR (^1^H, ^13^C, DEPT135, DEPT90) and 2D NMR spectra (1H, COrrelationSpectroscopY (COSY), Heteronuclear Single Quantum Coherence (HSQC), Heteronuclear Multiple Bond Correlation (HMBC), and Nuclear OverhausEr SpectroscopY (NOESY) were run with a direct probe head (5 mm PADUL 13C-1H Z-Grd). In order to increase sensitivity, TOtal Corelated SpectroscopY (TOCSY) NMR spectra was recorded with an inverse probe head (5 mm PHTXI 1H-13C/15N Z-Grd). Then, ^1^H spectrum calibration was performed by solvent pic (D_2_O) as internal reference (4.70 ppm). Chemical shifts (δ) are expressed in parts per million (ppm) and coupling constant (*J*) in Hz. All NMR experiments were carried out by using pulse sequences supplied by the spectrometer manufacturer (Bruker) 1D and 2D spectra. 

Proton NMR spectrum was acquired using 6 KHz Spectral Width (SW), 64 K complex data point, acquisition time (aq) of 0.87, relaxation delay (D1) of 2 s, number of scan (ns) of 30,000, number of dummy scan (ds) 32 and a 30° flip angle pulse width. Prior to Fourier transformation, the FIDs were multiplied by an exponential line broadening function of 0.3 Hz. ^13^C NMR spectrum was acquired using SW of 37.5 KHz, 64 K complex data point, aq of 5.45 s, D1 of 2 s, ns of 80, ds of 4 and a 30° flip angle pulse width. Then. 1H decoupling was achieved using WALTZ 16 pulse sequence. Prior to Fourier transformation, the FIDSs were multiplied by an exponential line broadening function of 2 Hz. DEPT (90,135) NMR spectrum were acquired using SW of 30KHz, 64K complex data point, aq of 1.09 s, D1 of 2 s, ns of 2000, ds of 32 and a 90° flip angle pulse width. 1H decoupling was achieved using WALTZ 16 pulse sequence. Prior to Fourier transformation, the FIDSs were multiplied by an exponential line broadening function of 1Hz.

gs-COSY spectrum was obtained with a SW of 6 KHz in both dimensions, 2K complex data point in F2, and 265 t1 increments (12 scan by increment) in F1, aq of 0.17 s, D1 1.5 s. Prior to Fourier transformation, a SINE window function (SSB = 0) was applied in both dimension and the data were zero filled and linear predicted (NC = 32) to 1K data points in F1.

gs-TOCSY phase sensitive (STATES–TPPI mode) using MLEV 17 pulse sequence for spin lock and WATERGATE 3-9-19 for water suppression was obtained with a SW of 7 KHz in both dimension, 2K complex data point in F2, 265 t1 increments (48 scans by increment) in F1, aq of 0.14 s, D1 2 s. MLEV 17 pulse sequence for spin lock was set to 100 ms. Water suppression was achieved using WATERGATE pulse sequence. Gradient pulse was sine shape (SINE 100), 1.5 ms long (p16) with 100 ms gradient recovery delay (D16) and strengths set to 20%. A 71.4 ms delay (D19) was used for binomial water suppression using Prior to Fourier transformation, a QSINE window function (SSB = 2) was applied in both dimension and the data were zero filled and linear predicted (NC = 32) to 1K data points in F1.

gs-NOESY phase sensitive (STATES–TPPI mode) was obtained with a SW of 6 KHz and 1K complex data points in F2, a SW of 21.3 KHz and 256 t1 increments (32 scans by increment) in F1, aq of 0.17 s, D1 2 s and a mixing time of 600 ms. Prior to Fourier transformation, a QSINE window function (SSB = 2) was applied in both dimensions and the data were zero filled and linear predicted (NC = 32) to 1K data points in F1.

gs-HSQC phase sensitive (echo-antiecho mode) was obtained with a SW of 6 KHz and 2K complex data point in F2, a spectral width of 21.8 KHz and 256 t1 increments (64 scan by increment) in F1. Other main parameters are 0.08 s for aq, 1.5 s for D1. Prior to Fourier transformation, a QSINE window function (SSB = 2) was applied in both dimension and the data were zero filled and linear predicted (NC = 32) to 1K data points in F1.

gs-HMQC (QF mode) was obtained with a SW of 6 KHz and 4K complex data point in F2, a spectral width of 28.9 KHz and 256 t1 increments (300 scan by increment) in F1. Other main parameters are 0.34 s for aq, 1.4 s for D1 and a mixing time of 125 ms (delay optimized for long range coupling of *J* = 8 Hz). Prior to Fourier transformation, a SINE window function (SSB = 0) was applied in both dimension and the data were zero filled and linear predicted (NC = 32) to 1K data points in F1.

### 4.5. Chemical Synthesis 

The synthetic peptide was generated on the 0.6 mmol scale by a manual solid-phase synthesis apparatus using conventional solid-phase peptide synthesis Fmoc/But procedure.

For this synthesis, we used Wang resin support preloaded with serine as the C-terminal, which is Fmoc-protected. The side-chain-protecting groups of the amino acids were Boc ether for Tryptophan (Trp), Lys and tert-butyl for Serine (Ser). The peptide was synthesized using the Fmoc method, according to a two-step procedure: (i) 15 min of protection steps using a mixture of 20% piperidine in DMF; (ii) coupling reactions performed with the protected amino acid diluted in DMF, using Bop as the coupling reagent in the presence DIEA for 1 h.

The completeness of each coupling reaction was monitored by means of the Kaiser test. If the test was positive, the coupling reaction was repeated using the BOP reagent in the presence of DIEA, with mixing for 1 h. The protected peptidyl resins were treated with a mixture of 95% TFA, 2.5% water, and 2.5% EDT for 1 h. After cleavage, the solid support was removed by filtration and the filtrate was concentrated under reduced pressure. The cleaved peptide was precipitated with diethyl ether and lyophilized. The purification and the identification of the synthetic peptide were assessed by analytical HPLC in the same condition of native peptide purification as described above. Unless otherwise noted, all reagents were purchased from Sigma-Aldrich commercial company.

### 4.6. In Silico Study

#### 4.6.1. Study of Pharmacokinetic and Pharmacological Properties

To better characterize the physicochemical and pharmacokinetic properties of the newly identified molecule, the SwissADME application (ADME for Absorption, Distribution, Metabolism and Elimination) was used to determine the liposolubility carcass and calculate the Log P*o*/*w* partition coefficient (*o*/*w* for octanol/water) (SIB, Swiss Institute of Bioinformatics© 2021, http://www.swissadme.ch/index.php, accessed on 5 December 2021).

#### 4.6.2. Sequence Similarity Search

The search for sequence homology was carried out with their alignment using NCBI BLASTp programs. The study of the similarity between the peptide sequences was carried out by means of the BLOSUM50 substitution matrix (BLOcks matrix for 50% identity calculated by BLASTp). Percent identity matrix was created by Clustal Omega.

#### 4.6.3. Homology Based Peptide-Protein Docking 

The complex of FPRL-1 with AaTs-1 was built based on a co-crystal template of the C5a receptor with its antagonist, the exapeptide PMX53 (Ace-Phe-[Orn-Pro-dCha-Trp-Arg]), using a comparative approach work-flow. We used target sequences of FPRL-1 (Uniprot accession code P25090) and AaTs-1 as the receptor and ligand, respectively. The template sequences (C5a and AaTs-1) were then aligned with their equivalent homologues (FPRL-1 and AaTs-1) target sequences using SALIGN routine from MODELLER. The alignment was used to guide the model building with the same program [65]. Fifty conformers were generated, from which we selected the one with the lowest DOPE score [66] and for which we established the Ramachandran plot to assess the stereochemical quality of the model. 

Because Modeller introduces spatial constraints derived from intramolecular and intermolecular interactions, it poorly models the flexibility of the peptide. Therefore, to get a low-resolution pose, we performed a peptide-protein docking using FlexPepDock method from Rosetta, which assumes that the starting pose is close to the global minimum and uses its enhanced sampling capacity to drive the structure of the peptide into its native bound conformation. The prediction starts from a pre-docked pose (the complex generated by homology modeling). The refinement engine consists of a Monte Carlo simulation in which 100 stages are affected in coarse grain low-resolution mode followed by high-resolution stages. This combination permitted the sampling of the backbone and the rigid body movements of the atom groups of AaTs-1. Another 100 high-resolution refinement stages were also run. The simulation results in 200 final complexes, which were then scored according to their Rosetta full atom energy, for which we analyzed the 10 best solutions.

### 4.7. Functional Characterization

#### 4.7.1. DNA Fragmentation

Cells were plated and treated or not treated with the native and the synthetic peptide at the IC_50_ (0.56 mM) for 72 h as described above for the proliferation assay. Staurosporin (STA) (1 µM) was used as a positive control. After washing with PBS, cells were detached with trypsin and centrifuged in PBS. Genomic DNA was extracted using kit genomic DNA miniprep (BIO BASIc INC, Markham, ON, Canada). DNA fragmentation was analyzed using 1% agarose gel electrophoresis, colored with ethidium bromide (BET). 

#### 4.7.2. Analysis of Apoptosis by Flow Cytometry

U87 cells (8 × 10^4^ cells/well) were treated or not treated with the synthesized peptide at the IC_50_ (0.56 mM) for 24, 48, and 72 h. STA at 1 µM was used as a positive control. The percentage of U87 cells undergoing apoptosis was determined by the FITC Annexin V Apoptosis detection Kit (BD Pharmingen^TM,^ San Jose, CA, USA) according to the manufacturer’s protocol. The kit utilizes a fluorescent dye (FITC) conjugated to Annexin-V to detect phosphatidylserine (PS) on the external membrane of apoptotic cells and the Propidium Iodide (PI), which passes through the cell membrane in the stage of necrosis or late apoptosis. Percentages of cells in early (AnV^+^/PI^−^) and in late stages (AnV^+^/PI^+^) of apoptosis were determined by a flow cytometer-based instrument (BD FACSCanto^TM^ II, San Jose, CA, USA) using the CellQuests software (Becton-Dickinson, Franklin Lakes, NJ, USA). Cells in necrosis were marked only with IP (AnV^−^/PI^+^).

#### 4.7.3. Cell Cycle Analysis 

Cells (8 × 10^4^ cells/well) were plated and treated with the native peptide or its synthetized form at IC_50_ (0.56 mM), which was applied as described above for the proliferation assay. After 72 h, cells were washed with PBS and resuspended in hypertonic buffer (20 mM HEPES; 0.16 M NaCl; 1M EGTA; pH 7.2; 0.05% triton 100x) 1 h at 4 °C. After centrifugation, cells were treated with ribonuclease (RNase; 100 μg/mL) for 15 min. Propidium Iodide (PI) (40 μg/mL in PBS) was then added and allowed to incubate for an additional 30 min in darkness. The DNA content was determined using a FACS flow cytometer (BD FACSCanto^TM^ II, San Jose, CA, USA). CellQuests software (BD, San Jose, CA, USA) was used to fit the data to a mathematical model for relating DNA content to the cell cycle stage. Cells with a DNA content between 2N and 4N were designated as being in the G1, S, or G2/M phase of the cell cycle. The number of cells in each compartment of the cell cycle was expressed as a percentage of the total number of counted cells.

#### 4.7.4. Western Blot Analysis

Cells treated or not treated with 0.56 mM of the native peptide, were lysed with Laemmli buffer (10 mM Tris; pH 6.8; 10% glycerol; 1% SDS). Protein extracts were collected by centrifugation at 10,000 rpm and quantified using the BCA assay kit (Bicinchoninic Acid Protein Assay kit, Sigma). Equal amounts of protein samples were separated by SDS–polyacrylamide gel electrophoresis (SDS-PAGE) and transferred to PolyVinyliDene difluoride (PVDF) membrane (Amersham^TM^ Hybond^TM^, GE Healthcare, Chicago, IL, USA). After incubation with primary and secondary antibodies (dilution 1:1000 and 1:5000 respectively) (Cell Signaling Technology^®,^ Beverly, MA, USA), the immuno-reactive proteins were detected by the enhanced chemi-luminescence detection system (ECL, X-OMAT EX II Developer and Replenisher, KODAK, Rochester, NY, USA).

#### 4.7.5. Intracellular Ca^2+^ Measurement

Cytosolic Calcium concentrations were studied using the ratiometric fluorescent dye Fura-2-AM from Thermo Fischer Scientific (Waltham, MA, USA). Approximately 5 × 10^4^ U87 cells were seeded in a 96-wells plate Coverglass Bottom. Then, 24 h later, cells were loaded with Fura2-AM (1 μM) for 45 min at 37 °C. Fluorescence acquisition (excitation 340 and 380 nm; emission 510 nm) was performed on the Flexstation 3 microplate reader (Molecular Devices, San Jose, CA, USA). Stimulation with 4 μM of Thapsigargin (TG)-induced ER calcium release was performed at 100 s of recording under calcium free conditions to determine the magnitude of intracellular calcium release. Next, 2 mM of CaCl_2_ were added at 500 s in order to quantify Store Operated Calcium Entry (SOCE). The magnitude of SOCE and intracellular release were estimated by ΔF/F_0_ with the exception of basal Ca^2+^ concentrations estimated as the average of initial F_340 nm_/F_380 nm_ values. Analyses were performed using SoftMax Pro Software (5.4.6 version, Molecular Devices, San Jose, CA, USA).

### 4.8. Image Processing

The images of cells in culture were taken by a digital high definition color Leica-MC 170 HD camera (SG) coupled to a *Leica Microsystems* optical microscope (Wetzlar, Germany) Ltd. The images were treated by Leica Application Suite (version 4.9.0).

All western blot transfer films were scanned by the Tiny Scanner application (version 4.0.2, developed by Appxy, HK, CN) without any color modification, using a HUAWEI GR5 2017 smartphone equipped with a 12 MP + 2 MP Dual-lens Rear Camera.

Band intensity quantification was applied equally to the several fields by ImageJ software (Bethesda, MD, USA) without changing the brightness or contrast.

### 4.9. Statistics

Statistical analysis, evaluated by one-way analysis of variance (ANOVA) with GraphPad Prism 6 (GraphPad Software, San Diego, CA, USA), was made using Student *t* test. Data are reported as mean ± SD. Statistical comparisons between three or more sets of data were performed. Differences were considered significant when *p* < 0.05.

## Data Availability

The protein sequence data will appear in the UniProt Knowledgebase under the accession number C0HLZ5. The RMN data has been deposited in BMRbig Biological Magnetic Resonance Data Bank under the title “AaTs-1: a tetrapeptide from Androctonus australis scorpion venom, inhibiting U87 glioblastoma cells proliferation by p53 and FPRL-1 up-regulations -NMR data” and the Entry ID bmrbig30 (https://bmrbig.org/released/bmrbig30) (accessed on 5 December 2021). The main NOESY and HMBC correlations have also been deposited with BMRbig Entry bmrbig31 (https://bmrbig.org/released/bmrbig31) (accessed on 5 December 2021).

## Supplementary Materials

The following are available online. Table S1: Effect of H9 on mice viability/Lethality test; Figure S1: Effect of H9 on U87 cell viability (**A**). Microscopic observation of U87 cells after treatment with MTT (**B**); Figure S2: Effect of H9 on U87 cell adhesion, migration and invasion; Figure S3: Sequence similarity search with AaTs-1; Figure S4: Genomic DNA (**A**), apoptosis (**B**) and cell cycle analysis (**C**).

## Sample Availability

Samples of the compounds are available from the authors.

## Abbreviations

Aa: *Androctonus australis*; AaTs-1: Tetrascorpin-1, AKT: Protein kinase; BCA: Bicinchoninic Acid; BSA: Bovine Serum Albumin; CH3CN: Acetonitrile; Coll-I: Collagen-I; ECM: Extracellular Matrix; Cltx: Chlorotoxin; EGFR: Epidermal Growth Factor Receptor; ERK: Extracellular-signal-Regulated Kinase; ESI-Q-TOF: Electrospray ionization quadrupole time-of-flight; FBS: Fetal Bovine Serum; Fg: Fibrinogen; Fn: Fibronectin; FPLC: Fast Protein Liquid Chromatography; FPRL-1: human formyl peptide receptor like-1; GBM: Glioblastoma multiforme; HPLC: High Pressure Liquid Chromatography; ICV: intracerebro-ventricular; JNK: Jun N-terminal Kinases; MEM: Moyen Essentiel Minimal; MMP matrix metalloproteases; MS: Mass Spectrometry; MTT: 3-(4-5 dimethylthiazol-2-yl)-diphenyltetrazolium Bromide; NMR: Nuclear Magnetic Resonance Spectroscopy; PBS: Phosphate Buffered Saline; Pl: Polylysin; PVDF: Polyvinylidene Difluoride; SDS: Dodecyl sodium sulfate; SOCE: store-operated Ca^2+^ entry; STA: Staurosporin; TMZ: temozolomide; TFA: Trifluoro Acetic Acid.
