# Supplementary material for: AaTs-1: A Tetrapeptide from Androctonus australis Scorpion Venom, Inhibiting U87 Glioblastoma Cells Proliferation by p53 and FPRL-1 Up-Regulations
Source: Molecules. 2021 Dec 15;26(24):7610. doi: 10.3390/molecules26247610 (PMC8704564; doi:10.3390/molecules26247610)
Supplement: Supplementary file 1 [file molecules-26-07610-s001.zip › molecules-1468062-supplementary.pdf]

Supplementary Materials

AaTs-1: a Tetrapeptide from *Androctonus australis* Scorpion Venom, Inhibiting U87 Glioblastoma Cells Proliferation by p53 and FPRL-1 Up-Regulations

Dorra Aissaoui-Zid [1,*], Mohamed-Chiheb Saada [1], Wassim Moslah [1], Marie Potier-Cartereau [2], Aude Lemettre [2], Houcemeddine Othman [1,3], Marc Gaysinski [4], Zaineb Abdelkafi-Koubaa [1], Soumaya Souid [5,6], Naziha Marrakchi [1], Christophe Vandier [2], Khadija Essafi-Benkhadir [5] and Najet Srairi-Abid [1,*].

[1] Université de Tunis El Manar, Institut Pasteur de Tunis, LR20IPT01 biomolécules, venins et application théranostique, 1002, Tunis, Tunisie.
[2] Université de Tours, INSERM, N2C UMR 1069, Tours, France.
[3] Sydney Brenner Institute for Molecular Bioscience, Faculty of Health Sciences, University of the Witwatersrand, Johannesburg, South Africa.
[4] Université Nice Sophia Antipolis, Plate-Forme Technologique de Chimie Service RMN, Faculté des Sciences, Parc Valrose, 06108 Nice, France.
[5] Université de Tunis El Manar, Institut Pasteur de Tunis, LR16IPT04 Epidémiologie Moléculaire et Pathologie Expérimentale appliquée aux Maladies Infectieuses, 1002, Tunis, Tunisie.
[6] School of Basic Pharmaceutical and Toxicological Sciences, College of Pharmacy, University of Louisiana at Monroe, 1800 Bienville Drive, Monroe, LA, 71201, USA.
* Correspondence: Aissaoui-Zid, Najet Srairi-Abid; Adress: Institut Pasteur de Tunis, 13 Place Pasteur Tunis, 1002, Tunis Belvédère Tunisia; Tel : + 216 71 844 688 (ext. 409); fax : +216 71 791 833; e-mail : aissaoui.dorra@yahoo.com; najet.abid@pasteur.rns.tn

Table S1. Effect of H9 on mice viability/Lethality test.

**Group**	**1**	**2**	**3**	**4**	**5**	**6**	**7**
Mice number	3	6	6	6	6	6	6
Administration	Intracerebro-ventricular injection						
Solution/injection	0.1% BSA in water (vehicle)					H9	in vehicle
Volume/injection				μL			5
Dose (μg)	0	1	1.5	2	2.5	3	3.5
Mortality	0/3	0/6	0/6	0/6	0/6	0/6	0/6

Figure S1

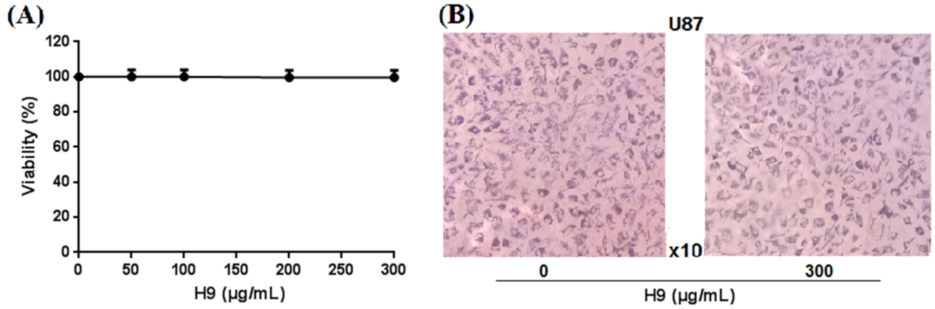

Figure S1. Effect of H9 on U87 cell viability (A). Microscopic observation of U87 cells after treatment with MTT (B). Cells were incubated with different concentrations of the tested molecule. After 24 h, cells were treated with MTT (0.5 mg/mL). The crystals formed after the reduction of MTT by mitochondrial dehydrogenases were dissolved with DMSO. The quantification of live cells was achieved by measuring absorbance at 560 nm. A negative control was used in the same condition with mock-treated cells. Cells treated with 0.1% Triton X-100 were used as positive control.

Figure S2

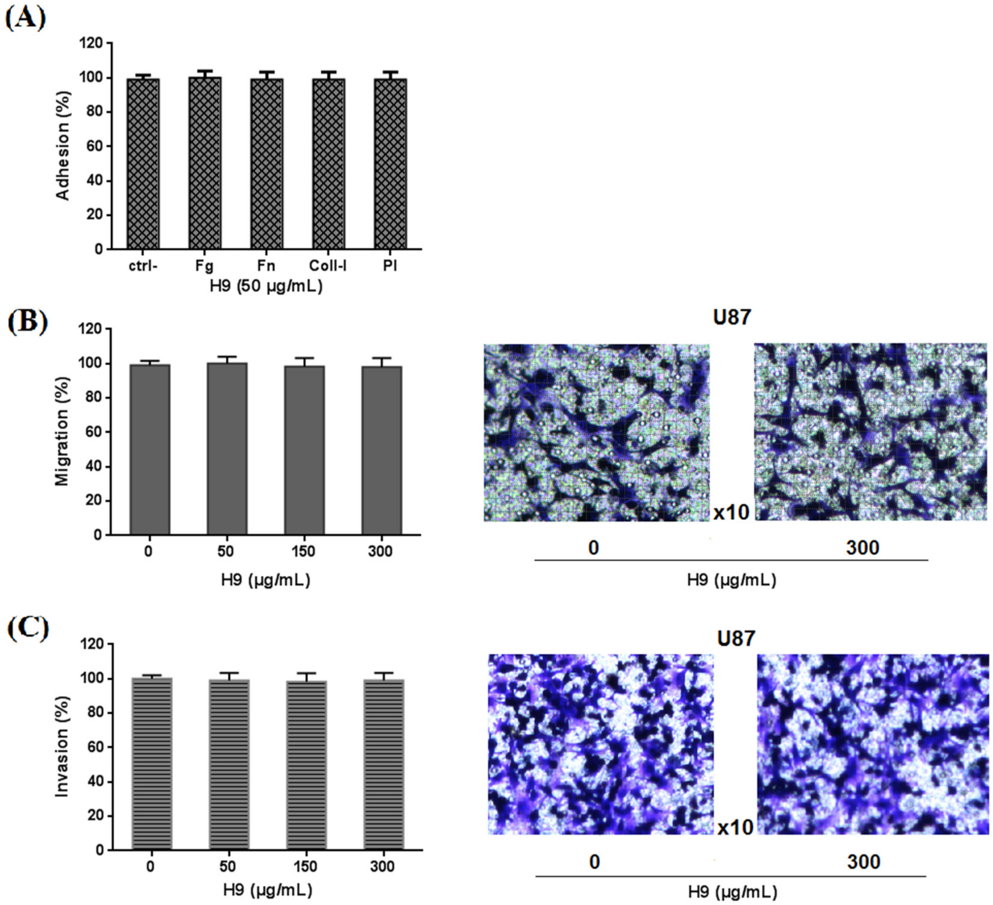

Figure S2. Effect of H9 on U87 cell adhesion, migration, and invasion.**(A) Adhesion assay:** Cells treated or not treated with H9 were deposited on wells coated with fibronectin (Fn) at 10 µg/mL, fibrinogen (Fg) at 5 µg/mL, collagen-I (Coll-I) at 50 µg/mL, as extracellular matrix (ECM), or poly-L-lysine (Pl) at 20 µg/mL. Cells were allowed to adhere to the substrata for 2 h at 37 °C. **(B) Migration**

assay: Cells pretreated with H9 were added to pre-coated membranes with Fn (5 µg/mL), for 2 h at 37 °C, and allowed to migrate for 5 h at 37 °C in Boyden chambers.**(C) Invasion** assay: Matrigel™ was added on the membrane in Boyden chambers and allowed to solidify for 3 h at 37 °C. U87 cells pretreated with H9 were then added and incubated for 22 h at 37 °C. After incubation, attached cells were fixed, stained by 0.1% crystal violet (**Microscopic observation to the right**), then lysed with 1% SDS and quantified by measuring absorbance at 560 nm.

Figure S3

AaTs-1	I**WK**S---	4
Tetrapandin-2	L**WK**T---	4
FPRL-1 ligand	-**WK**YMVM	6

Figure S3. Sequence similarity search with AaTs-1.(A) Multiple sequence alignment by BLAST. AaTs-1: Tetrascorpin from Aah; Tetrapandin-2 from *Pandinus imperator*; WKYMVm FPRL-1-ligand: a FPRl-1 synthetic peptide ligand.

Figure S4

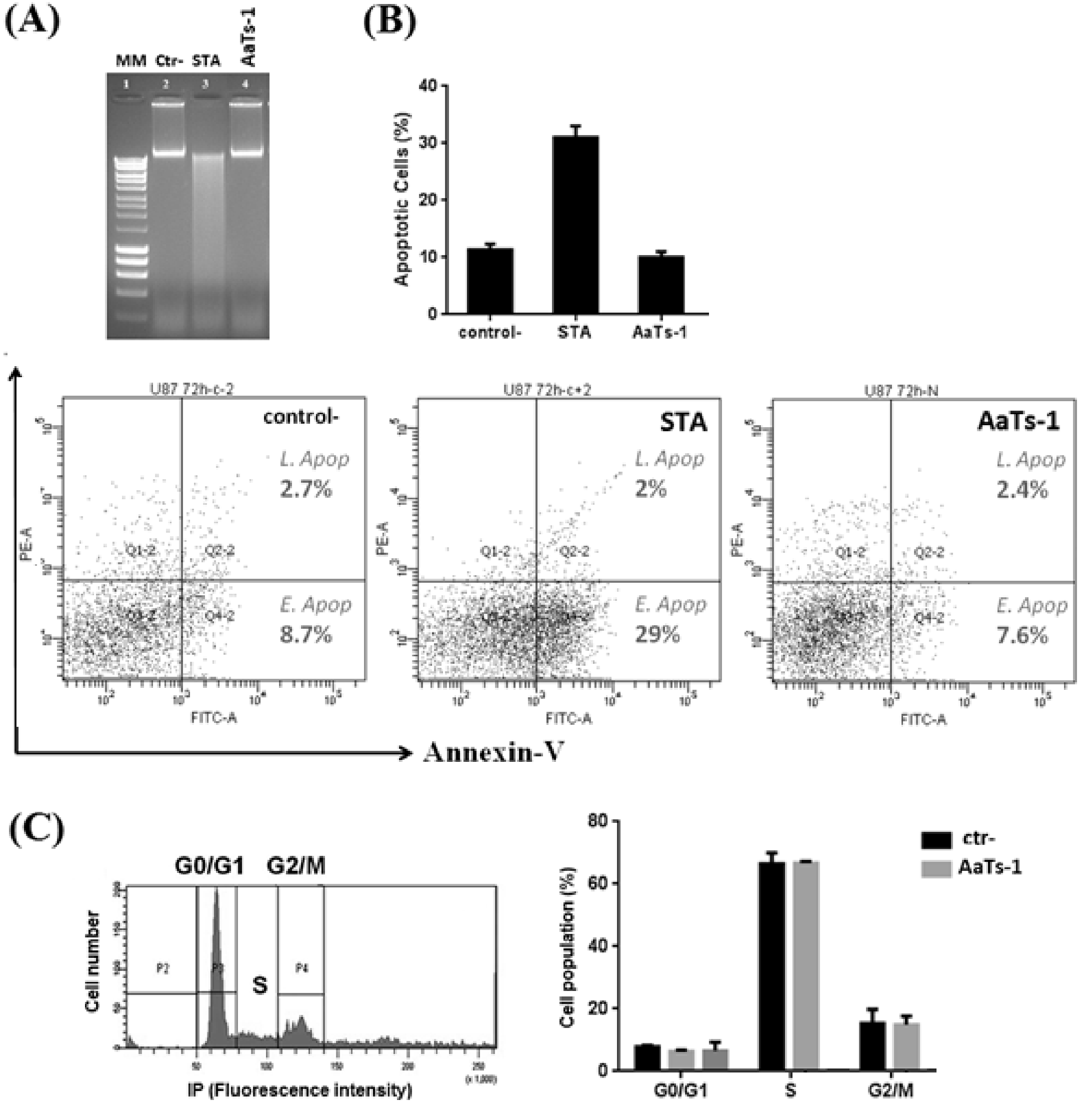

Figure S4. Genomic DNA, apoptosis, and cell cycle analysis. U87 cells were treated for 72 h with 0.56 mM of AaTs-1. For each FACS experiment, 10,000 events were analyzed. **(A)** Gel electrophoresis of genomic DNA of U87 cells treated with AaTs-1 (0.56 mM). MM: Molecular marker, STA: Staurosporin, E. Apop: Early apoptosis, L. Apop: Late apoptosis. **(B)** Quantitative analysis of apoptotic cells determined by FACS measurement. **(C)** Effect of AaTs-1 on the progression of the U87 cell cycle.
